# Sleep Quality in Patients with Heart Failure in the Spanish Population: A Cross-Sectional Study

**DOI:** 10.3390/ijerph17217772

**Published:** 2020-10-23

**Authors:** Pablo Jorge-Samitier, Angela Durante, Vicente Gea-Caballero, Isabel Antón-Solanas, María Teresa Fernández-Rodrigo, Raúl Juárez-Vela

**Affiliations:** 1 Department of Physiatry and Nursing, University of Zaragoza, Hospital Clínico Lozano Blesa, Avda. San Juan Bosco, 15, 50009 Zaragoza, Spain; pjorge@salud.aragon.es; 2 Department of Biomedicine and Prevention, University of Rome Tor Vergata, Via Montpellier, 1 00133 Rome, Italy; angela.durante@uniroma2.it; 3 Nursing School La Fe, Adscript Center of University of Valencia, Research Group GREIACC, Health Research Institute La Fe, 46026 Valencia, Spain; 4 Department of Physiatry and Nursing, University of Zaragoza, Research Group GENIAPA, Calle Domingo Miral s/n, 50009-Zaragoza, Spain; ianton@unizar.es; 5 Department of Physiatry and Nursing, University of Zaragoza, Calle Domingo Miral, s/n, 50009 Zaragoza, Spain; 6 School of Nursing, University of La Rioja, Research Group GRUPAC and Research Institute IDI-Paz, C/Duquesa de la Victoria 88, 26004 Logroño, La Rioja, Spain; raul.juarez@unirioja.es

**Keywords:** heart failure, sleep disorder, sleep quality

## Abstract

*Background*: Heart failure is a major problem in western societies. Sleep Disorders maintain a bidirectional relationship with heart failure, as shown by studies conducted in other countries. This study aims to describe the quality of sleep in Spanish patients with heart failure. *Materials and methods*: We carried out a cross-sectional study to analyze the quality of sleep in a sample of 203 patients with a diagnosis of heart failure admitted to an Internal Medicine Service. The Pittsburg Sleep Quality Index (PSQI) was used to evaluate sleep quality in our sample over a one-month period. *Results*: 75% of the sample presented sleep disorders. The most common problems included the interruption of sleep (73.5% nocturia and 30% breathing difficulties); 35% had poor sleep efficiency; 33% showed a decrease in daytime performance; 84% had used hypnotics at some point to induce sleep and 35% used them regularly. *Conclusions*: This is the first study to report on the perceived sleep quality of patients with heart failure in Spain. Self-perception of sleep quality differed from that estimated by the PSQI. The prevalence of the use of sleep-inducing medication was very high. The diurnal dysfunction generated by sleep disorders in a heart failure environment can contribute to the development of self-care and cognitive deterioration problems.

## 1. Introduction

Heart failure (HF) is a pathophysiological disorder in which the heart is unable to respond to the body’s metabolic demands, or it does it at the expense of increased ventricular filling pressure [1].

HF is one of the largest public health problems worldwide, and its complex and progressive nature usually leads to the loss of quality of life, a high rate of hospital re-admissions, and high mortality [2,3,4]. In Spain, HF affects 6.8% of the general population and its incidence increases with age (8% in people between 65% and 75%, and 16.1% among those over 75) [3,4,5]. This is mainly due to the increased life expectancy of patients with acute coronary syndrome, advances in secondary prevention, advances in the treatment of the disease and the progressive aging of the population [4,5].

The progression of heart failure is characterized by decompensations that require medical attention and that often lead to hospital admission [6]. This leads to an increase in the cost of health resources (between 1.8–3.1% of the total public health budget) [3,4,5], especially in the elderly population, with an average length of stay of ±9.5 days [5,6].

One of the consequences of HF is sleep disorders (SD) [2,3,4,5,6,7]. SD affect more than 75% of patients with HF and result in difficulty in inducing or maintaining sleep, waking up too early and not being able to fall asleep again, and excessive drowsiness [8,9]. According to Zuurbier et al. [10], SD are mainly due to certain clinical manifestations such as orthopnea, nocturia, and restless leg syndrome, and are associated with older age and worse survival rates [10,11]. The worsening quality of sleep affects physical health, cognitive performance, daily activity, mental health, and has cardiac consequences in patients with HF [10,11]. In addition, previous studies have found an association between SD and a reduction in adaptation skills [12], attention problems, decreased memory, poor adherence to treatment and self-care difficulties [13,14,15,16,17]. Specifically, in patients with HF, SD contribute to the development and progression of cardiovascular disease, poor quality of life and higher levels of depressive symptoms [8,11,12,15,16,17].

An incorrect approach to SD in patients with HF causes complications in the medium to long term clinical evolution [9]. The association between sleep-disordered breathing and cardiovascular pathology (hypertension, HF, ischemic cardiopathy) is well documented in the literature. As a result, possible symptoms related to sleep apnea in cardiac patients are increasingly being identified in the anamnesis by clinicians. For example, addressing sleep-disordered breathing by means of positive airway pressure is part of the treatment and provides better results in the evolution of heart disease [18,19,20,21]. Research on sleep problems in patients with HF in Spain has focused on sleep-disordered breathing such as apnea. Previous studies [22] analyzed the relationship between apnea and HF in the Spanish population. However, no previous studies were published on the quality of sleep perceived by patients with HF in Spain. In addition, the Spanish sociocultural context is different from other sociocultural contexts where similar investigations have been carried out, including Northern Europe, Asia, and America, which may result in a variation in the sleep pattern of patients who have HF. Furthermore, Spain is one of the countries with the highest life expectancy in the world. We argue that knowledge of the factors associated with poor sleep quality can contribute to the development of higher quality and individualized care in this population.

Research into the perceived quality of sleep, especially in local populations, can help contextualize available evidence and identify different perspectives from which to launch interventions aimed at improving the health of patients with HF, delay hospital re-admissions, minimize hospital stays, and thereby reduce health spending. Therefore, the aim of this study is to describe the quality of sleep in Spanish patients with HF and to identify those aspects that negatively affect their health outcomes and that are modifiable.

## 2. Materials and Methods

We performed a cross-sectional study to determine the quality of sleep in a convenience sample of 203 patients admitted to a large Spanish hospital due to decompensation of HF. All of our participants were admitted to the internal medicine ward of the Hospital Universitario Lozano Blesa (University Hospital Lozano Blesa). This non-probabilistic sampling technique allowed as to recruit a large sample of participants over a short period of time. Hospital-based recruitment was preferred to outpatient clinic and primary care due to the grouping of cases. Considering that the Pittsburg Sleep Quality Index (PSQI) measures the quality of sleep perceived in the previous month at home, we argue that our recruitment technique did not interfere with our findings.

The inclusion criteria to participate in our study were: (1) Being admitted to hospital due to decompensated HF; (2) Scoring > 2 in the Six Item Screener (SIS); (3) Voluntarily accepting to participate in the study and signing the consent form.

The SIS is a simple, yet reliable tool used to identify patients with cognitive impairment in healthcare [23]. It consists of three questions related to temporary orientation (day of the week, month, and year) and three questions that measure the patient’s ability to remember and repeat three words.

Sociodemographic data were collected including age, gender, level of study, number of previous hospitalizations, and grade of HF according to the New York Heart Association (NYHA) classification (Table 1).

The Charlson comorbidity index (CCI) predicts the ten-year mortality for a patient who may have a range of comorbid conditions and was used to identify the presence of other pathologies in our sample [24]. It includes, in addition to the patient’s age, 26 items or medical conditions scored from 1–6 with a total score calculated from 0 to 37, giving a total comorbidity score (Table 2).

We used the PSQI to analyze the quality of sleep in our sample over a one-month period. This tool combines quantitative and qualitative information that discriminates against subjects as “good and bad sleepers” and allowed us to evaluate the factors that can affect the quality of sleep. The PSQI evaluates seven aspects of sleep: subjective quality, latency, duration, usual efficiency, disorder, use of sleeping medications, and daytime dysfunction. The final score is the sum of the scores in each of the seven constructs and ranges from 0 to 21 (the higher the score, the worse the sleep quality). A score above 5 indicates poor sleep quality and below 5 good sleep quality [2] (Table 3).

The interviews were conducted between 1 January and 31 December 2017, after the patients gave informed consent to participate. All interviews were conducted by the same researcher within the internal medicine service of the Hospital Clínico Universitario Lozano Blesa of Zaragoza. This study was approved by the Ethics and Clinical Research Committee of Aragon (CEICA) (ID P115/2016). The SPSS version 21 program was used for statistical analysis (Version 21 for Windows, IBM Corp., Armonk, NY, USA). According to the aims of this study, we used descriptive statistics (mean and standard deviation) for demographic and frequency data.

## 3. Results

A total of 203 participants (102 women and 101 men) took part in this study. The mean age was 81.1 years (SD 8.69). The majority of patients were Spanish nationals (99.5%), were married or widowed (45.3% and 46.8% respectively) and were educated to primary school level (87.2%). Approximately 72% of our participants shared their home with others (71.9%) and declared that they had what they needed to live on (76.4%). In terms of lifestyle habits, the majority of our patients were non-smokers (92.6%) and did not consume any alcohol (93%) (Table 1).

Table 2 summarizes the disease burden of our population. The most frequent diseases associated with HF were: high blood pressure (82.4%) and atrial fibrillation (69.4%). Just over a third of our patients suffered from anemia (34.3%), diabetes (35.2%), moderate or severe kidney disease (37%) and/or previous acute myocardial infarction (36.1%). 12.3% of our patients were obese. Only 5.6% were diagnosed with sleep apnea syndrome. Finally, less than 5% of patients presented peripheral vascular disease, dementia, connective tissue disease, mild, moderate, or severe liver disease, cancer, and HIV.

Most of the subjects interviewed attended a specialized service for chronic follow-up of HF (77.3%), while 21.2% were treated in primary care. Regarding the time since diagnosis, 83.6% of our patients had been diagnosed 2 to 5 years before. Functional class was NYHA II or III (49.3% and 48.5% respectively) for the vast majority of our sample. The results of the PSQI are collected in Table 3.

The frequency of occurrence of each disorder is considered “occasional” when it appears less than once a week, “habitual” when it appears 1–2 times a week, and “continuous” when it appears three times or more per week.

With regard to the first four components (quality, duration, latency, and efficiency), approximately 30% of our patients took more than 30 min to fall asleep; 60.4% slept more than 6 h. In addition, 53.2% of our sample spent more than three-quarters of the time they were in bed sleeping. Interestingly, 81% of our patients described their sleep quality as “pretty good or very good,” while the Pittsburgh test classified 73.3% of our sample as “bad sleepers”, with overall scores above or equal to 5 with an average of 9.3 ± 3.35 (SD). In terms of component 5, sleep disturbances, most patients woke up one or more times during the night or too early (61.5%); 73.5% got up one or more times to go to the bathroom. About one-third of our participants were unable to fall asleep within the first half hour one or more times a week, and 29.8% experienced some respiratory distress, of whom 9% did so frequently. Similarly, around 29% reported coughing, snoring and having nightmares on a regular basis, and 9% on an ongoing basis; 11.2% had pain regularly and 3.7% on an ongoing basis.

Component 6 measures the use of hypnotic medication. 82.4% of our patients had hypnotic drugs in their home to fall asleep and admitted to using them on an occasional, regular, or continuous basis. Specifically, 35% had used them once or several times in the previous week.

One of the direct effects of SD is daytime dysfunction, as measured in component 7, characterized by drowsiness or low mood that makes it difficult to perform social or everyday activities such as eating or driving. 35% experienced this type of problems frequently or continuously, whereas 17% had problems doing these activities enthusiastically.

**Figure 1:**
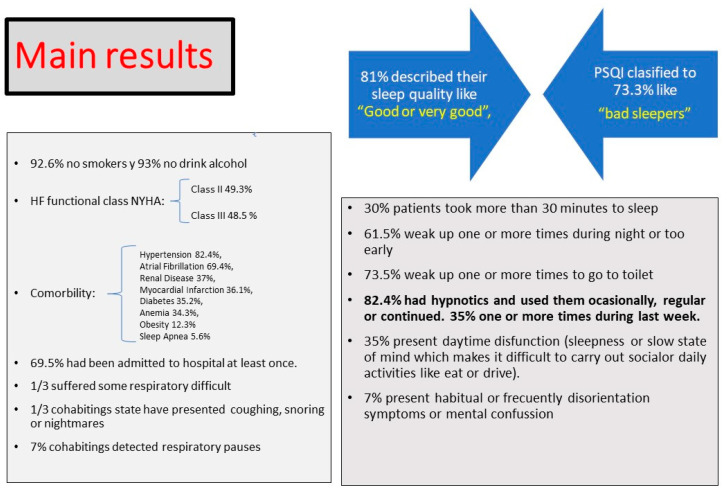
Main Results.

The results of the PSQI for the persons cohabiting with a patient with HF are presented in Table 4. Approximately 50% of the respondents did not share a house or bed with anyone, so they could not answer the subsequent questions, and only a third shared a room and/or bed with a patient with HF. After adjustment, our results indicate that only 7% of the cohabitants detected breathing pauses during sleep in a habitual or frequent manner in the previous month. Twenty-seven percent reported strong snoring once or more times a week, 8.5% heard teeth brushing while sleeping and 7% observed symptoms of disorientation or mental confusion in a habitual or frequent way. A summary of the main results is presented in Figure 1.

## 4. Discussion

The population included in this study reflects the current profile of HF patients admitted to an internal medicine service of a large tertiary hospital in Spain. Most of our participants were older adults with a variable number of comorbidities, especially cardiovascular diseases such as hypertension, atrial fibrillation, anemia, diabetes, nephropathy, y and acute myocardial infarction. It is expected that by 2050, Spain will be country with the highest percentage of old people in the world [25]. It is, therefore, not surprising that the average age of our population (81.2 years) was significantly higher than in previous studies (ranging from 62 to 74 years) [16,17]. This may also explain why most of our patients had functional class II and III HF, while other studies reported a majority of patients with NYHA class II HF.

One of the factors most frequently associated with poor sleep quality and cardiovascular problems is obesity. Specifically, a correlation has been found between obesity and obstructive sleep-disordered breathing [26,27]. Interestingly, while our participants’ BMI revealed a high rate of obesity in our population (45.6%), only 12.3% of our patients had been formally diagnosed and this information had been collected in their clinical history. Furthermore, this figure is well below the national average for obesity in persons aged 60 or over (30%) [28]. Thus, our findings suggest that obesity is being underdiagnosed in our sample of patients with HF. This, naturally, would have consequences on the patients’ experience of SD as well as on the healthcare professionals’ ability to determine their likely cause. For example, according to Oxilia Estigarribia [29], 40% of patients with HF experienced central apnea and 11% had obstructive apnea. However, only 5.6% of our patients were formally diagnosed with sleep apnea in our study.

SD are common in the population of older adults. According to Torres et al. [30], between 12–40% of the population over 65 years of age experience SD. Previous studies [12,13,14,15,16,17] analyzing the association between HF and SD in different sociocultural contexts suggest a significantly higher prevalence of SD in the population older adults with HF (63–91%). This is despite the fact that the average age of our population was higher than in previous studies. The association between sleep apnea and HF is yet to be studied in our country. The cause of this discrepancy may be attributed to the underdiagnosis of sleep apnea in our population. We were unable to contrast this information with the results from the PSQI for the patients’ cohabitants, as only one third of our participants shared their house and/or bed with a relative and, therefore, the generalizability of these results is limited.

Nearly three quarters of our participants were classed as bad sleepers according to the PSQI. Our results are similar to those obtained in previous studies [16,17], with percentages ranging from 63% to 91.2%. We observed a discrepancy between the patients’ perceived quality of sleep and their actual sleep quality. Similar results were obtained by Kyoung Suk Lee et al. [11] and Moradi et al. [12]. As was suggested by Harvey et al. [31], this apparent discrepancy between objective and subjective appraisals of patients’ sleep quality highlights the complexity of this construct and the importance of understanding the subjective meaning of sleep quality.

With regard to the time that our patients took to fall asleep, we observed notable differences between our findings and those reported in previous studies [2,11,16]. Specifically, whereas only 30% of our patients took more than 30 min to fall asleep, approximately 75% of the participants in previous studies took more than 30 min to fall asleep.

Approximately 30% of our population consumed sleep-inducing medication. Our results are similar to those reported by Kyoung Suk Lee et al. [11]. However, there is great variability in the use of sleep-inducing medication in our population. Santos et al. [2] reported a prevalence in the use of sleep-inducing medication of 9.5% in Brazil, two studies in China [17] and Taiwan [32] reported a prevalence of 14%, and Moradi et al. {12] reported a prevalence of 20% in the use of hypnotics in Iran. There may be multiple reasons for this variation, from a degree of information bias to differences in the prescription and management of this medication. The most commonly used drugs in SD therapy are no longer barbiturates, which generated high-impact side effects such as tolerance and dependence. At present, they have been replaced by short and medium half-life benzodiazepines and benzodiazepine receptor agonists. However, these prescription drugs are not entirely free from complications and side effects related to tolerance and dependence [33,34,35,36]. Cognitive behavioral therapies that have longer-term positive effects and without the side effects of drugs are also recommended [37]. Frequent use of sleep-inducing drugs in our context reflects that SD is prevalent in the population of Spanish patients with HF. However, continuous use of sleep-inducing medication can be a problem during HF decompensation. This is important as, during hospital admission, chronic treatment is generally modified, which may result in the temporary suspension of hypnotic medication and consequent increased risk of side effects.

In terms of sleep disturbances, it is noteworthy that 73.5% of our patients said that they got up to go to the bathroom at least once or more a week. These findings coincide with those reported in similar studies [2,9,11,16,17]. Improper use of diuretics may explain these figures [16].

The sum of the aforementioned disturbances may be the cause of subsequent daytime dysfunction, which contributes to cognitive decline, depressive symptoms, lack of treatment adherence, memory impairment, and self-care problems [11,12,13,14,15]. This has an impact not only on the fact that patients with HF struggle to stay active during the week [2,12,17], but also on the fact that they experience difficulty detecting signs of decompensation of their disease and making decisions about their treatment [15].

## 5. Limitations

Since this is a descriptive study, we have not used the statistical projection to infer associations. We would also like to highlight that data collection took place during hospital admission to an internal medicine ward in a large hospital. Although the PSQI measures the quality of sleep perceived in the previous month at home, it is possible that our results may have been influenced by the patients’ actual sleep quality at the time of data collection.

## 6. Conclusions

This is the first study of the perceived quality of sleep in the Spanish population with HF. In our population, over 70% of patients with HF had poor sleep quality according to the PSQI.

We found a lack of correlation between perceived sleep quality and sleep quality as measured by the PSQI in our population. This difference could be explained by the number of hours that patients stayed in bed without sleep, namely 35% did not sleep well before 30 min and/or experienced disruptions in their sleep (getting up to urinate, symptoms such as dyspnea or cough). This could account for more than a third of the sample using sleep-inducing medication on a regular or frequent basis. It is well-known that the presence of daytime dysfunction due to SD is related to self-care problems and cognitive impairment. We found that a just over a quarter of our population used sleep-inducing medication to fall asleep. Future studies should investigate the prevalence in the use of hypnotics in the Spanish population and study the association between SD and the use of sleep-inducing medication. In addition, subsequent investigations should look into the therapies currently used to control sleep disorders in our context, as well as their effects on the patients’ quality of life and impact on the evolution of HF. Additionally, we recommend that future studies analyze the correlation between SD and hospital admission of patients with HF due to decompensation. This will be crucial to design and implement interventions aimed at preventing SD and HF-related complications.

## Figures and Tables

**Table 1:** Sociodemographic characteristics.

	Items	*N* (%) ± SD
Sex	Male	102 (50.2)
	Female	101 (49.8)
Age		81.1 ± 8.7
Marital Status	Single	15 (7.4)
	Married	92 (45.3)
	Divorced	1 (0.5)
	Widower	95 (46.8)
Study Level	Primary School	177 (87.2)
	Secondary School	13 (6.4)
	Vocational Education and Training	1 (0.5)
	University	6 (3)
Place of residence	Zaragoza	165 (81.3)
	Other	38 (18.7)
Nationality	Spanish	202 (99.5)
	Other	1 (0.5)
Occupation	Employee	3 (1.5)
	Freelance	5 (2.5)
	Unemployed	2 (1)
	Retired	193 (95.1)
Persons at home	0	57 (28.1)
	1	94 (46.3)
	2	32 (15.8)
	3	14 (6.9)
	4	2 (1)
	Religious community	4 (2)
Children		2.4 ± 1.68
Economic autonomy	More than enough to live on	39 (19.2)
	Enough to live on	155 (76.4)
	Difficult to live on	9 (4.4)
Smoking	Yes	13 (6.4)
	No	188 (92.6)
Alcohol Drinking	Yes	12 (5.9)
	No	189 (93,1)
Height (m)		1.47 ± 45.2
Weight (Kg)		77.5 ± 16.6
BMI (Kg/m)		28.9 ± 5.6
Normal		69 (34.3)
Overweight		41 (20.4)
Obesity		91 (45.3)
Hospital admissions in the last month	0	62 (31)
	1	98 (49)
	2	22 (11)
	3	14 (7)
	4 or more	4 (2)
New York Heart Association HF Functional Class	I	1 (0.7)
	II	66 (49.3)
	III	65 (48.5)
	IV	2 (1.5)
Years since diagnosis	1	6 (3)
	2	29 (14.3)
	3	22 (10.8)
	4	34 (16.7)
	5	22 (10.8)
	>5	14 (11.7)
Cause of HF	Ischemic	33 (25.6)
	Non ischemic	94 (72.9)
	Idiopathic	2 (1.6)
Type of HF follow-up assistance	Hospital	157 (78.1)
	Primaria Care	43 (21.4)
	Others	1 (0.5)

**Table 2:** Charlson Comorbidity Index (CCI).

Items	*N* (%)
Myocardial infarction	39 (36.1)
Congestive heart failure	108 (100)
Peripheral vascular disease	1 (0.9)
Cerebrovascular disease	8 (7.4)
Alzheimer’s disease/dementia	1 (0.9)
Chronic pulmonary disease	24 (22.2)
Connective tissue disease	3 (2.8)
Peptic ulcer disease	6 (5.6)
Mild liver disease	1 (0.9)
Moderate or severe liver disease	1 (0.9)
Diabetes	38 (35.2)
Diabetes with end organ damage	9 (8.3)
Hemiplegia	1 (0.9)
Moderate or several renal disease	40 (37.0)
Any solid organ tumor	13 (12.0)
Obesity	13 (12.3)
Metastatic solid organ tumor	1 (0.9)
AIDS	1 (0.9)
Lymphoma	0 (0.0)
Leukemia	2 (1.9)
Atrial fibrillation	75 (69.4)
Sleep apnea	6 (5.6)
Hypertension	89 (82.4)
Anemia	37 (34.3)
Pulmonary hypertension	16 (14.8)
Another disease	96 (88.9)

**Table 3:** Pittsburg Sleep Quality Index.

	Items	*N* (%) ± SD
Classification		
Good sleepers		50 (26.7)
Bad sleepers		137 (73.3)
Score		
Global		7.5 ± 4.1
Classified as bad sleepers		9.3 ± 3.35
Component 1. Subjective sleep quality	Very good	110 (59.1)
	Pretty good	41 (22)
	Pretty bad	24 (12.9)
	Very bad	11 (5.9)
Component 2. Sleep latency		
How long does it take you to fall asleep?	0–15 min	72 (35.5)
	16–30 min	56 (29.9)
	31–60 min	38 (20.3)
	>60 min	21 (11.2)
Component 3. How many hours do you sleep?	>7h	93 (45.8)
	6–7h	20 (9.9)
	5–6h	44 (21.7)
	<5h	30 (14.8)
Component 4. Sleep efficiency		
(hours asleep/hours stayed in bed) %	>85%	60 (32.6)
	75–84%	38 (20.7)
	65–74%	35 (19)
	<65%	51 (27.7)
Component 5. Sleep disturbances		
Get up in the middle of the night or get up too soon	Never in the last month	52 (27.8)
	Less than once/week	20 (10.7)
	1–2 times/week	49 (26.2)
	3 or more times/week	66 (35.3)
You have to get up to go to the bathroom	Never in the last month	28 (15)
	Less than once/week	15 (8)
	1–2 times/week	59 (31.6)
	3 or more times/week	85 (45.5)
Do not breathe comfortably	Never in the last month	78 (41.7)
	Less than once/week	57 (30.5)
	1–2 times/week	35 (18.7)
	3 or more times/week	17 (9.1)
Loud coughing or snoring	Never in the last month	97 (51.9)
	Less than once/week	38 (20.3)
	1–2 times/week	36 (19.3)
	3 or more times/week	16 (8.6)
Feel too cold	Never in the last month	172 (92)
	Less than once/week	12 (6.4)
	1–2 times/week	3 (1.6)
	3 or more times/week	0
Feel too hot	Never in the last month	172 (92)
	Less than once/week	9 (4.8)
	1–2 times/week	5 (2.5)
	3 or more times/week	1 (0.5)
Nightmares	Never in the last month	132 (70.6)
	Less than once/week	25 (13.4)
	1–2 times/week	22 (11.8)
	3 or more times/week	8 (4.3)
Pain	Never in the last month	127 (67.9)
	Less than once/week	32 (17.1)
	1–2 times/week	21 (11.2)
	3 or more times/week	7 (3.7)
Other reasons		16 (7.9)
Component 6. Use of hypnotic medication	Never in the last month	33 (17.6)
	Less than once/week	88 (47.1)
	1–2 times/week	50 (26.7)
	3 or more times/week	16 (8.6)
Component 7. Daytime dysfunction		
How many times have you felt tired to drive, eat or do social activities in the last month?	Never in the last month	115 (61.5)
	Less than once/week	8 (4.3)
	1–2 times/week	8 (4.3)
	3 or more times/week	56 (29.9)
How many problems have you felt to maintain enthusiasm for performing tasks in the last month?	Never in the last month	127 (67.9)
	Less than once/week	27 (14.4)
	1–2 times/week	20 (10.7)
	3 or more times/week	13 (7)

**Table 4:** Pittsburg Sleep Quality Index for the cohabitant.

Categories	Items	*N* (%)
Do you share the house or bed with anyone?	I do not share a house or bed	92 (49.2)
	I share a house with someone who sleeps in another room.	24 (12.8)
	I share a house with someone who sleeps in the same room but different beds	20 (10.7)
	I share a bed with someone	50 (26.7)
Presents strong snoring?	Never in the last month	50 (70.4)
	Less than once/week	2 (2.8)
	1–2 times/week	11 (15.5)
	3 or more times/week	8 (11.3)
Has breathing pauses while sleeping?	Never in the last month	59 (83.1)
	Less than once/week	7 (9.9)
	1–2 times/week	4 (5.6)
	3 or more times/week	1 (1.4)
Can you perceive a jaw rubbing while sleeping?	Never in the last month	63 (88.7)
	Less than once/week	2 (2.8)
	1–2 times/week	4 (5.6)
	3 or more times/week	2 (2.8)
Has had a time of disorientation or confusion?	Never in the last month	63 (88.7)
	Less than once/week	3 (4.2)
	1–2 times/week	3 (4.2)
	3 or more times/week	2 (2.8)
Has had some other signs of restlessness while sleeping?	Never in the last month	63 (8.7)
	Less than once/week	3 (4.2)
	1–2 times/week	3 (4.2)
	3 or more times/week	2 (28)
